# Protecting optimal childhood growth: systematic nutritional screening, assessment, and intervention for children at risk of malnutrition in the Kingdom of Saudi Arabia

**DOI:** 10.3389/fnut.2024.1483234

**Published:** 2024-11-07

**Authors:** Robert D. Murray, Sanaa Y. Shaaban, Mohammed Al Amrani, Wajeeh Aldekhail, Faisal A. Alhaffaf, Abdulaziz O. Alharbi, Ali Almehaidib, Yasir Al-Suyufi, Muath Al-Turaiki, Ahmed Amin, Mohammed Y. Hasosah, Musa Alkhormi, Ziyad T. Mirza, Rola Sleiman, Ghassan Sukkar

**Affiliations:** ^1^Department of Pediatrics, The Ohio State University College of Medicine, Columbus, OH, United States; ^2^Faculty of Medicine, Ain Shams University, Cairo, Egypt; ^3^Division of Gastroenterology, Department of Pediatrics, Sulaiman Al Habib Medical Services Group, Riyadh, Saudi Arabia; ^4^Department of Pediatrics-Gastroenterology Section, King Faisal Hospital and Research Center, Riyadh, Saudi Arabia; ^5^Department of Pediatrics-Gastroenterology Division, Prince Sultan Military Medical City, Riyadh, Saudi Arabia; ^6^Pediatric Gastroenterology Department, King Saud Hospital Ministry of Health, Riyadh, Saudi Arabia; ^7^Department of Pediatrics, King Faisal Specialist Hospital and Research Center, Riyadh, Saudi Arabia; ^8^Department of Pediatrics, King Saud Hospital, Unaizah, Qassim, Saudi Arabia; ^9^Pediatric Department, King Salman Hospital, Ministry of Health, Riyadh, Saudi Arabia; ^10^Abbott Laboratories, Dubai, United Arab Emirates; ^11^Pediatric Gastroenterology Department, King Abdullah International Medical Research Center, Ministry of National Guard Health Affairs, Jeddah, Saudi Arabia; ^12^Pediatric Gastroenterology Division, King Saud Medical City, Dallah Namar Hospital, Riyadh, Saudi Arabia; ^13^Division of Pediatric Gastroenterology, Department of Pediatrics, King Fahd Armed Forces Hospital, Jeddah, Saudi Arabia; ^14^Al Habib Medical Group, Al Rayyan Hospital, Riyadh, Saudi Arabia; ^15^Department of Pediatrics, King Saud Bin Abdulaziz University for Health Sciences, Jeddah, Saudi Arabia

**Keywords:** diet quality, mid-upper arm circumference (MUAC), pediatric, risk assessment, screening, undernutrition, *z*-score

## Abstract

**Background:**

In 2024, the Kingdom of Saudi Arabia Advisory Board on Pediatric Nutrition (KSA-ABPN) reviewed childhood undernutrition in the Middle East. We sought to foster efficient nutritional care for infants and children at nutritional risk. Severe malnutrition due to starvation is rare in Saudi Arabia, so we focused on early recognition and treatment of children with mild growth impairment that forewarns risk for further nutritional decline. This paper summarizes our findings and introduces a recommended guide for nutritional screening, assessment, and follow-up interventions.

**Objective:**

The KSA-ABPN aimed to build an algorithm with pathways and tools to facilitate up-to-date nutrition-care practices for infants and children. The algorithm is intended to encourage consistent professional training-for and use-of validated tools, adoption of standardized thresholds for intervention, and delivery of nutritional support. Consistent care will increase opportunities for comparative analyses of various treatment strategies and their health and cost outcomes.

**Recommendations:**

We developed a 4-stage algorithm for identifying and caring for children at nutritional risk: (i) screening for clinical risk factors and age-related growth measures, (ii) observation of malnutrition-related physical signs, diet history, and/or laboratory detection of evidence indicating specific nutrient deficiencies, (iii) assessment of the severity of nutritional deficit, and (iv) development of a patient-specific Nutrition Care Plan that includes diet counseling, supplementation, routine monitoring, and follow-up.

**Conclusions:**

By helping professionals identify nutritional risk and specific nutritional deficits in infants and children early in the clinical course, we seek to expand quality nutritional care and ensure that children grow and develop fully.

## 1 Introduction

Malnutrition is defined as an imbalance between nutrient requirements and intake (1). This definition includes traditional not only underweight, wasting, and stunting, but also overweight and obesity, and micronutrient insufficiencies—conditions that reflect poor diet quality. In infants and children, undernutrition leads to a cumulative deficit of energy or protein intake, with or without sufficient intake of specific micronutrients. Such shortfalls can negatively affect growth and development as well as engender adverse clinical and social outcomes (2). Among infants and children worldwide, malnutrition remains a critical threat to health and wellbeing (3).

In May of 2024, the Kingdom of Saudi Arabia Advisory Board on Pediatric Nutrition (KSA-ABPN) met to address the problem of childhood undernutrition in the Middle East. Specifically, the Board aimed to raise awareness of the signs representing inadequate nutrition early in its course to prevent the serious complications of moderate-to-severe malnutrition. The Board recommended nutritional evaluation by (i) screening for and identifying those at nutritional risk, (ii) identifying and treating underlying medical problems, and (iii) ensuring the adequacy and completeness of dietary intake as early and as efficiently as possible.

### 1.1 Early-life nutrition matters

Consistent nutrition in infancy and childhood is essential for each child to grow and reach his or her full potential for lifelong function, wellbeing, and achievement (4–9). Specifically, adequate early-life nutrition is needed to support physical growth, such as bone length, muscle mass, and tissue refinement. Nutrition promotes the structure and function of organs, such as the gastrointestinal tract, the immune system, the cardiorespiratory system, and the kidneys (4, 7). Importantly, healthful nutrition in the first 3 years of life is key to supporting synaptogenesis and myelination in the rapidly developing brain and nervous system (10–13).

The consequences of undernutrition in infants and children are wide-ranging and often life-long, including poor health outcomes as well as high health-economic costs (14). Malnutrition in infants and children has been associated with developmental or intellectual delay, poor school performance, muscle weakness or loss, vulnerability to infections and delayed wound healing, immune dysfunction, and prolonged hospital stays and higher costs of care (6–8, 13, 15). Children with severe undernutrition and stunted growth may experience adverse consequences in adulthood, i.e., increased likelihood of lower energy expenditure, fat accumulating in the central-body region, insulin resistance with elevated risk of developing type 2 diabetes, chronic conditions of hypertension and dyslipidemia, and lowered cognitive and work capacity (13).

### 1.2 Contributing causes of poor nutrition in infancy and childhood

The most common cause of acute undernutrition is having limited access to nutrient-rich foods (16). Pediatric malnutrition accompanies other social determinants of health, such as economic instability and poor access to healthcare and other social support (17). Secondary causes of malnutrition can be related to disease, including infections (18), severe acute conditions such as cancer and traumatic injury or burns (2, 19, 20), or chronic conditions such as autoimmune inflammatory disorders (21), congenital heart disease, and diseases of the kidneys, liver, or intestine (2, 22, 23). Even where food supplies are sufficient, young children may be at risk for poor nutrition and growth due to selective eating behaviors (24).

### 1.3 Pediatric undernutrition: growth impairment and growth faltering

Undernutrition affects about one in five children younger than 5 years-old worldwide (25, 26). Inadequate intake of energy, protein, and micronutrients critical for growth leads to growth impairment (27). Serious nutritional shortfall is evidenced as growth faltering—*stunting* (the failure to achieve potential length or height for a particular age) or *wasting* (the failure to achieve appropriate weight-for-height due to weight loss or failure to gain weight) (27, 28). Stunting affected 22% of children under 5 years or 148 million worldwide in 2022, while wasting affected nearly 7% or 45 million (3). Despite recent progress, childhood undernutrition persists as a global challenge, including in the Middle East and Northern Africa (29–33). Further gains will require more concerted screening and earlier intervention.

### 1.4 International public health organizations agree on goals for eliminating childhood malnutrition

Through the United Nations (UN), the world pledged in 2015 to achieve Sustainable Development Goals (SDG) (34). These goals, also supported by the World Health Organization (WHO) and the Word Bank, included the ambitious target of eliminating all forms of malnutrition by 2030, i.e., *Zero Hunger* (34, 35). Undernutrition in infants and young children is an important target of the 2030 *Zero Hunger* goal (3). For children under 5 years-old, intermediate goals were set for 2025 to assess progress on reducing stunting (height for age < -2 standard deviations from the median of the WHO Child Growth Standards) and wasting (weight for height < -2 standard deviations from the median of the WHO Child Growth Standards) (36). These targets were to reduce by 40% the number of children under five who are stunted and to reduce childhood wasting to < 5% (37). Progress reports for SDG targets on child malnutrition in the Near East and Northern Africa indicate that only a few of the 22 countries are likely to meet the stunting and wasting goals (38). Saudi Arabia is progressing toward these goals (38). In Saudi Arabian children, severe malnutrition due to starvation is rare. Stunting and wasting among children under 5 years has dropped dramatically over the past two decades to < 4% (39). More directed actions are especially needed to meet overall healthy growth goals, so we now aim to lower undernutrition risk by focusing on early recognition and treatment of children with mild growth impairment or other signs that forewarn of nutritional decline or undernutrition.

## 2 KSA-ABPN algorithm development

Pediatric clinicians are uniquely positioned with multiple opportunities to ensure optimal dietary outcomes by recognizing nutritional problems in the 1st years of a child's life. These professionals can actively guide parents through transitions from breastfeeding to addition of complementary foods, expansion of the diet for food diversity, and shaping dietary habits of the child and family.

The KSA-ABPN members recognized that early identification of undernutrition or its risk is key to preventing serious consequences later for children in the Middle East. The KSA-ABPN leadership meeting was organized by Abbott Middle East and Northern Africa (Abbott MENA, Dubai, UAE) and took place on May 31, 2024, in Riyadh, Saudi Arabia. Two groups participated—a 12-member KSA-Advisory Board and two global presenters. All participants had expertise in gastroenterology, pediatrics, and nutrition. Robert D Murray, MD (USA) and Sanaa Youssef, MD (Egypt), opened the meeting by reviewing the medical literature and practices for pediatric nutrition screening, diagnosis of undernutrition and its causes, and treatments by age, cause, and severity of nutritional deficits. They also reviewed current strategies for care. KSA-ABPN participants then created, debated, and refined a pediatric nutrition care algorithm to guide healthcare professionals for nutrition screening, assessment, and follow-up nutrition care. The algorithm was designated *A Process to Screen for Malnutrition Risk, Make Diagnoses, and Provide Supportive Treatment*. Following this meeting, a draft of the manuscript was submitted to KSA-ABPN members for final review. All KSA-ABPN were polled to confirm consensus agreement on the final content.

## 3 New Saudi algorithm

Infants and young children are at particularly high risk for nutrient-related adverse health consequences due to high nutrient needs in support of rapid growth and development. Even though Saudi Arabia has witnessed rising prosperity, some families remain at socioeconomic risk, while other children show nutritional risk due to picky eating behaviors as they transition from breastfeeding to complementary foods to family diets (24).

**Figure 1:**
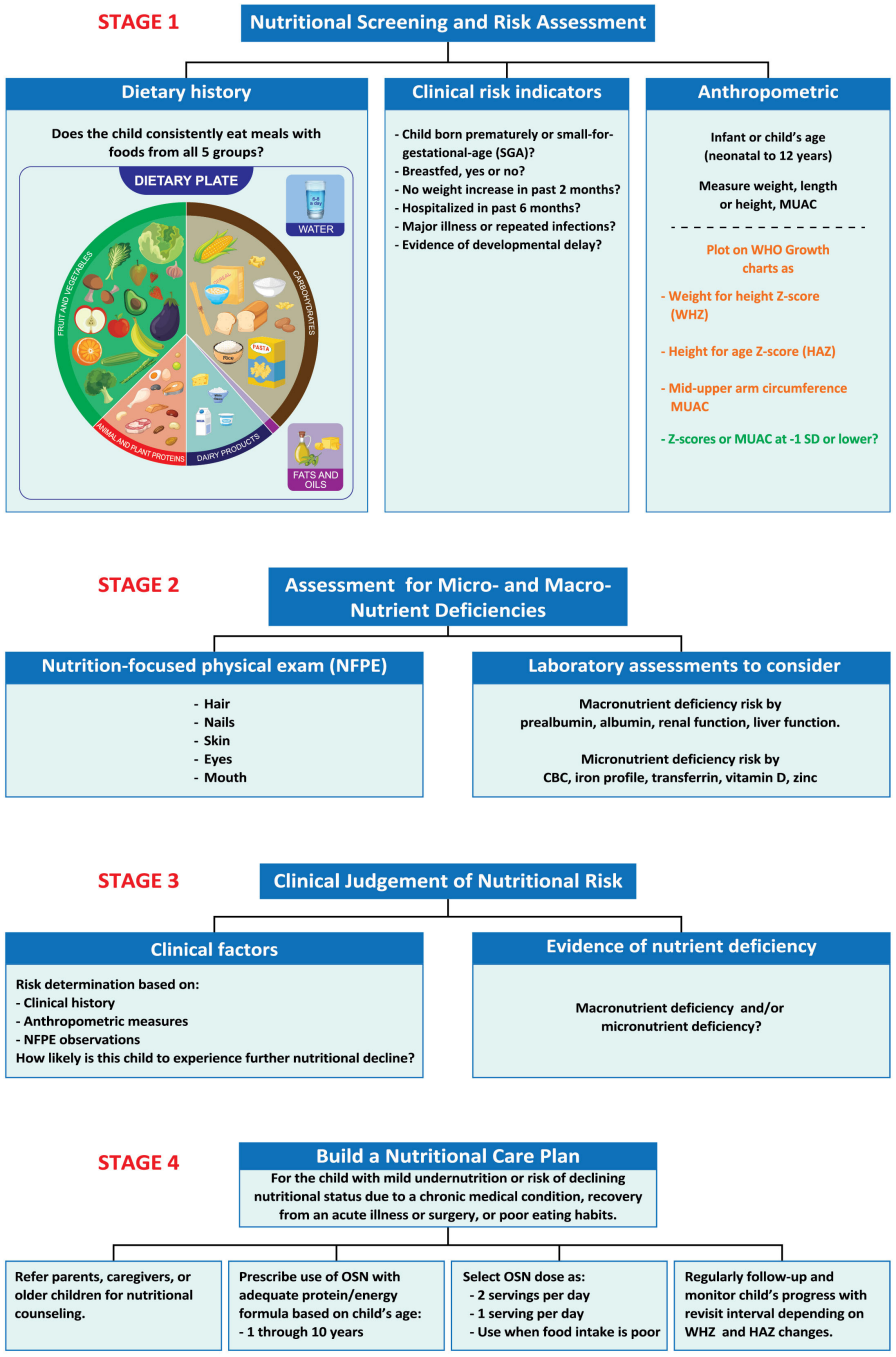
Stages 1–4. A process to screen for malnutrition risk, make diagnoses, and provide supportive treatment. Stage 1. Identify clinical risk factors and age-related growth measures that indicate increased risk for undernutrition. Stage 2. Observe malnutrition-related physical signs and/or conduct laboratory measures to detect evidence indicating specific nutrient deficiencies. Stage 3. Use clinical judgment to put together prior observations to make a clinical diagnosis of undernutrition, if present, and decide upon treatment based on likelihood of nutritional decline. Stage 4. Develop a patient-specific Nutrition Care Plan that includes routine monitoring and follow-up. CBC, Complete blood count; HAZ, height-for-age *z*-score; MUAC, mid upper-arm circumference; SGA, oral nutritional supplement, small for gestational age; WHZ, weight-for-height *z*-score.

Screening and assessment represent different processes. Screening identifies potential nutritional problems, while assessment builds support for diagnosis of malnutrition. Multiple tools can be considered for screening and assessment of pediatric nutrition status (14, 40). The KSA-ABPN developed a 4-stage algorithm to provide a simple, but thorough, systematic approach to nutritional screening, assessment and care (Figure 1).

### 3.1 Stage 1

The algorithm is relevant for infants and children up to 12 years old. For Stage 1, the algorithm advises pediatric clinicians to begin by collecting information about a child's risk for undernutrition, i.e., age, dietary habits; clinical risk indicators (prematurity, small-for-gestational age, SGA, failure to breastfeed) (41, 42); recent hospitalizations; diagnoses of acute or chronic diseases (16, 22, 43); experiencing repeated infections, especially those requiring use of antibiotics (18). The algorithm recommends querying about the child's dietary history, either by using a 3-day food diary or by asking whether the child eats widely from all five food groups—fruits, grains, vegetables, protein foods, and dairy.

The World Health Organization (WHO), along with the United Nations Children's Fund (UNICEF), recommend that children be exclusively breastfed for the first 6 months of life (44). Fewer than half of infants under 6 months old are exclusively breastfed worldwide (44). In the Arabic world, rates of breastfeeding are particularly low, with fewer than 20% of infants in Saudi Arabia being exclusively breastfed (45). Breastmilk is safe, nutritionally beneficial, and helps protect the infant against many common illnesses. Breastmilk provides energy and nutrients to meet the needs for the 1st months of life, and it continues to meet about half of a child's nutritional needs during the second half of the 1st year, and up to one third during the 2nd year of life (44). Human milk also contains bioactive compounds such as microRNA that may help regulate lipid and glucose metabolism, gut maturation, neurogenesis, and immunity (46). Importantly, breastmilk also supports healthy colonization of the infant gut (47). By contrast, not being breastfed has been associated with negative consequences such as higher rates of infections, elevated risks for sudden infant death syndrome in infancy, and later development of types 1 and type 2 diabetes (42).

Developmental delay is yet another factor associated with risk for poor nutritional status, although the relationship can be complex and bidirectional. For example, developmental delay predisposes children to nutritional deficits (48), while severe acute malnutrition predicts developmental delay (49).

The algorithm specifically recommends that pediatric clinicians use anthropometric measurements of weight, length/height, and determinations of *z*-scores according to the child's age and sex. *Z*-scores with negative values represent growth impairment and are an indicator of pediatric malnutrition. We further recommend taking measures of mid-upper arm circumference (MUAC). MUAC measures are simple screening methods with good sensitivity and accuracy, which can be adopted for community-based screening for nutritional risk in school-aged children, including adolescents (20, 23, 50–53).

Charts for determining z-scores for infants children up to 2 years are available via a World Health Organization (WHO) website (36). For boys and girls 2–19 years, the Centers for Disease Control (CDC) charts are used (54). To compare the measures with median anthropometric scores for infants and children of the same age and sex, *z*-scores represent standard deviations (SD) from the median, with negative z-scores reflecting values below the median. The weight-for-height *z*-score (WHZ) is an indicator of wasting, while length/height-for-age z-score (HAZ) can identify stunted growth or its risk. *Z*-scores between −2 and −3 indicate moderate malnutrition, while *z*-scores below −3 suggest severe malnutrition (40, 55). Children in these categories require prompt and thorough treatment. A Nutrition Care Pathway for the moderately-to-severely undernourished child was previously recommended (ASPEN guidelines); this plan includes documentation of signs, severity (mild, moderate, and severe) and etiology (illness or non-illness related), build a care team and make intervention plan with enteral or parenteral nutrition, or oral supplement nutrition (OSN, also termed oral nutritional supplement), as appropriate, and monitoring the child regularly following the diagnosis (56).

*Z*-scores between −1 and −2 represent children who appear to be mildly undernourished and who may be at risk of worsening nutritional status. All children with *z*-scores lower than −1 SD warrant further evaluation. Many will need treatment. Other children may be of nutritional concern due to micronutrient insufficiencies if their food intake or diet quality is poor. These are the children who are the main target of the KSA-ABPN Nutrition Care Plan.

### 3.2 Stage 2

In Stage 2, we suggest ways to identify possible macronutrient and micronutrient deficiencies. Current practice favors the use of laboratory markers only as a complement to a diet history and a thorough nutrition-focused physical examination (NFPE) (43). The NFPE assesses hair for dullness, thinness, or loss and examines skin for pallor, dryness, or dermatitis as indicators of poor nutritional status (43). Nutrition-related nail abnormalities may include spoon-shapes and dull, mottled, or pale appearance (43). Eye history review and examination may reveal night blindness, dull/dry sclerae or inner lids, or dull appearance of the cornea. Signs of poor nutrition in the mouth include dry or cracked lips, bleeding gums, darkened red appearance of the tongue, and presence of excessive tooth cavities (43).

Laboratory testing can be used to identify specific macro- and micronutrient deficiencies. Poor nutritional status may be indicated by low levels of albumin or prealbumin proteins; prealbumin is preferred over albumin because its shorter half-life reflects rapid changes of the nutritional state (57). However, using albumin and prealbumin as biomarkers of nutritional status (especially protein status) are more strongly influenced by inflammation than by nutrition (57). Markers of liver and kidney function are sometimes used to inform nutritional risk due to the importance of these organs to overall health. Common micronutrient deficiency can be detected, such as iron deficiency anemia (low complete blood count, low iron measures, or low levels of transferrin saturation), or low levels of vitamin D or zinc.

### 3.3 Stage 3

Stage 3 is an important step in which the clinician applies his or her judgment to weigh the evidence for risk of undernutrition based on medical history, anthropomorphic measures with *z*-scores, clinical signs and symptoms observed in the NFPE, diet history, and laboratory measures to diagnose undernutrition. If undernutrition is apparent, this stage reviews etiology (illness or non-illness related, specific micronutrient deficiency) and determines the level of severity or risk for nutritional decline. As discussed for Stage 1, *z*-scores indicate undernutrition severity as high (*z*-score < −3 SD), moderate (*z*-score between −3 and −2 SD), and mild forms (*z*-scores between −2 and −1 SD), recognized by growth impairment based on anthropometric measures and SD from the median value by age and sex. To facilitate decision-making, clinicians are urged to use as many data points as possible (over time and for different measures) (40). For further insight, the clinician may also use the Modified STAMP screening tool to assess risk of undernutrition, developed and validated for use in primary healthcare clinics in a community setting (58, 59).

### 3.4 Stage 4

Stage 4 focuses on the development of a treatment plan, often called a Nutrition Care Plan (NCP), for children who appear to be either mildly malnourished (−1 to −2 anthropometric z-score) or at risk of undernutrition due to micronutrient deficiencies or poor diet quality. The NCP is based on the clinician's judgment of the child's risk for nutritional decline and the child's estimated energy and protein needs, compared with his or her intake. Nutritional needs depend on the child's age and sex, as well as the estimated quantity of his or her nutritional shortfall. Generally stated, a child who is mildly undernourished meets only 51–75% of his or her protein/energy needs (40). The risk for undernutrition exists at any level of shortfall of energy and protein intake < 100% of what is needed. As well, specific micronutrient deficiencies (iron, vitamin D, calcium) may require supplemental doses of the nutrient. For most children with mild undernutrition or with risk for nutritional decline, the KSA-ABPN recommends nutritional counseling along with the use of a complete pediatric OSN along with as follows:

#### 3.4.1 Nutritional counseling

A referral to a registered dietitian or nutrition counselor can help parents or other caregivers or the older child implement a diet that is nutritionally sound and diverse. Education about diet diversity is an important feature of this step.

#### 3.4.2 OSN with age-appropriate nutrient density

The selected OSN should be formulated to provide age-appropriate levels of protein/energy and micronutrients, e.g., young child formulation for toddlers and young children (ages 1–10 years).

#### 3.4.3 OSN dose

Prescribed doses of OSN are based on clinical judgment that considers the severity of nutritional shortfall, the adequacy and diversity of foods in the child's usual diet and expected growth rates for the child's age and sex. Doses to consider are two servings per day, 1 serving per day, or supplementation on days when the child's amount or quality of food intake is poor. A meta-analysis by Zhang et al. included 11 pediatric studies of children (2–5 years) with mild undernutrition (anthropometric *z*-scores −1 to −2 SD below the mean); nearly all of the studies used two servings of OSN per day (450 kcals), some for as long as 48 weeks (60). Use of up-age OSN formulas is advised for children over 10 years because these formulas have higher levels of protein, key vitamins (A, D, E, and K) as well as calcium, iron, and zinc and other micronutrients.

#### 3.4.4 Follow-up and monitoring of nutritional status and growth

OSN should be continued until WHZ and HAZ both reach a plateau. Healthcare professionals need to schedule regular follow-up visits to the clinic for reassessment of the child until the child shows consistent improvement in diet quality and growth; the time interval between visits is determined by the severity of undernutrition or its risk, and by observations of catchup growth. Supplements can be weaned once catch-up growth is fully established based on WHZ and HAZ scores.

## 4 Discussion

### 4.1 Why it is important to identify and address poor nutritional status early in a child's life

Complications of undernutrition in infancy and early childhood are fully treatable and even preventable with early recognition. The KSA-ABPN algorithm will encourage pediatric clinicians to address nutritional risks promptly, lessening the likelihood of poor health outcomes. Early-life nutrition supports physical growth (bones, muscles, and tissues), promotes development and function of body systems (the gastrointestinal tract, the immune system, the cardiorespiratory system, and the kidneys (4, 7). Optimal nutrition in the first 3–5 years of life is key to supporting the rapidly developing brain and nervous system (10–13). On the other hand, if malnutrition in childhood goes untreated, a serious toll on human capital can result, as shown by slower cognitive development, reduced schooling attainment, and adult incomes decreased by up to 50% (15, 61). The responsibility for meeting UN and WHO Goals to lower childhood malnutrition rates (34, 35) can be supported by well-trained pediatric healthcare providers. In measurable terms, complete catch-up growth of both weight and height can only be achieved by improving the child's diet quality.

The KSA-ABPN algorithm was created to be simple, practical, and efficient. Recently published recommendations that were developed by a global consensus panel served as a foundation, providing a definition of faltering growth, an overview of clinical causes, and a guide to how faltering growth should be assessed and managed (62). The global group defined growth faltering as “a fall in WFA *z*-score of ≥1.0 that occurs over a period of 1 month or more and does not include the first 2 weeks after birth,” while catch up growth was defined as “increased growth velocity following recovery from illness or starvation…a physiologic increase in WFA z-score after a period of growth faltering.” A child with early evidence of mild undernutrition or its risk can usually be treated with dietary advice and ONS therapy. Unfortunately, such treatment may be overlooked when clinicians are not fully trained for appropriate prescription of ONS in children and adolescents at nutritional risk (63).

### 4.2 The benefits of pediatric nutritional supplements for patients at-risk

Numerous trials on use of pediatric OSN as part of treatment for children with or at risk of faltering growth have been identified in recent systematic reviews and meta-analysis (60, 64). A review by Cawood et al. (64) summarized results of ten trials that compared outcomes for children who received OSN vs. control. In more than 1,000 children (mean age 5 years) on OSN or controls, those who received OSN had significantly greater gains in weight and height; compliance with OSN was very high (98%) (64). These reviewers also reported an association between OSN use and reduced infections. In another systematic review and meta-analysis, researchers summarized the evidence from 11 trials of OSN intervention effects on growth of children (9-months to 12-years of age) who were undernourished or at nutritional risk but not yet malnourished (60). They found that in children with mild undernutrition. OSN use resulted in significantly better growth outcomes when compared to control treatments such as placebo treatment, usual diet, or dietary counseling alone (60).

## 5 Conclusions and clinical implications

This consensus statement sought to expand the reach of quality nutritional care by addressing gaps in nutritional knowledge and surveillance (8). As reported in this publication, members of the KSA-ABPN developed a 4-stage algorithm for identifying children with mild undernutrition or its risk. The algorithm guides determination of nutritional status and recommends nutritional care based on a child's (i) clinical history and age-related growth measures, (ii) observation of malnutrition-related physical signs and laboratory detection of evidence indicating specific nutrient deficiencies, (iii) a healthcare professional's clinical judgment for identifying malnutrition risk and determining its level of its severity, and (iv) building and adhering to a patient-specific Nutrition Care Plan that includes routine monitoring and follow-up.

### 5.1 Future directions

Despite global advances in economics, agriculture, and medicine, impaired growth due to undernutrition or specific micronutrient deficiencies in infancy and childhood remains a problem today. These conditions are most likely to threaten children in countries with low- to middle-income populations and in countries where food supplies have been disturbed due to wars or natural disasters (28, 33, 65).

While economic stability is essential to lowering levels of childhood malnutrition, further strategies are also needed (66) to meet the childhood growth-related targets set by the UN in 2015 (34, 35, 38). Childhood undernutrition occurs in every country in the Middle East, including Saudi Arabia (67–69). To eradicate undernutrition by 2030, Elmighrabi et al. called for multiple actions in the Middle East and Northern Africa; they specifically advised actions to prioritize maternal health and nutrition, invest in struggling families, and customize interventions to meet specific needs of each child in each country (31).

The KSA-ABPN anticipates that collaboration with pediatric specialists and community providers in other Middle East countries will generate experience using *A Process to Screen for Malnutrition Risk, Make Diagnoses, and Provide Supportive Treatment* in clinical practice settings.
